# Correction: Lin et al. SPE–UPLC–MS/MS for Determination of 36 Monomers of Alkylphenol Ethoxylates in Tea. *Molecules* 2023, *28*, 3216

**DOI:** 10.3390/molecules30071547

**Published:** 2025-03-31

**Authors:** Qin Lin, Yujie Qin, Hezhi Sun, Xinru Wang, Mei Yang, Xinzhong Zhang, Li Zhou, Fengjian Luo

**Affiliations:** Tea Research Institute, Chinese Academy of Agricultural Sciences, Hangzhou 310008, China; linqin202302@163.com (Q.L.); yujieq2020@163.com (Y.Q.); sunhezhi@tricaas.com (H.S.); wangxinru@tricaas.com (X.W.); maryyang1987@foxmail.com (M.Y.); zxz.1982@163.com (X.Z.)

Following publication, concerns were raised regarding the peer-review process related to the publication of this article [1]. Adhering to our standard procedure, the Editorial Board conducted an investigation which determined that, while the peer-review process did comply with MDPI’s Editorial process policy (https://www.mdpi.com/editorial_process), the contribution of one of the three reviewers did not comply with MDPI’s Guidelines for Reviewers (https://www.mdpi.com/reviewers#_bookmark11) and the expectations of the Editorial Board. As a result, the Editorial Board has decided to remove the contribution of one of the reviewers from the open peer-review record (https://www.mdpi.com/1420-3049/28/7/3216/review_report) and conduct a post-publication peer-review to add a new review report.

Based on the new review report, the authors have made the following revisions to this article [1]:

In order to improve the readers’ understanding of this article, some language descriptions and grammar, as well as the layout of some chapters, have been modified. The authors state that the scientific conclusions are unaffected. This correction was approved by the Academic Editor. The original publication has also been updated.

**Figures Correction**

Figures 1–5 were modified in the original publication [1]. 

**Addition of a Section**

The first paragraph of Section 2.3 and Figure 5 in the original publication [1] have been transferred to the new addition, Section 2.1. Due to these changes, the order of some Sections and Figures has been adjusted accordingly.

**Text Correction**

The sentence “The LODs of OPEOs and NPEOs are 0.008–2.09 μg/kg and 0.053–1.67 μg/kg, respectively.” has been added to the Abstract and Section 2.4, paragraph 2.

The sentence “In addition, the chemical structures of APEOs are related and very similar, as presented in Table S1.” has been added to Section 1, paragraph 2.

The sentence ”Quantitatively calculated oligomers of NPEOs and OPEOs in the sample are based on these alkylphenol ethoxylate standards.” has been added to Section 2.1.

**Supplementary Materials**

Table S1 (Supplementary Materials) has been added based on the reviewer’s suggestions. The sentence “Table S1. Chemical structures NPEOs and OPEOs.” has been added to Supplementary Materials. 

The sentence “Table S1: Mass spectrometric parameters for monitoring OPEO3–20 and NPEO3–20; Table S2. Validated parameters of APEO3–20 in spiked tea samples.” has been changed to “Table S2: Validated parameters of APEO3–20 in spiked tea samples; Table S3. Mass spectrometric parameters for monitoring OPEO3–20 and NPEO3–20.”

**References**

As recommended by one of the reviewers in the original publication [1], references 3 and 33 have been removed. In addition, due to the addition of Section 2.1 and changes to the order of paragraphs, some references have been adjusted accordingly.
