# Supplementary material for: SPE–UPLC–MS/MS for Determination of 36 Monomers of Alkylphenol Ethoxylates in Tea
Source: Molecules. 2023 Apr 4;28(7):3216. doi: 10.3390/molecules28073216 (PMC10096240; doi:10.3390/molecules28073216)
Supplement: Supplementary file 1 [file molecules-28-03216-s001.zip › molecules-2281710-supplementary.pdf]

## Supplementary Material

# SPE-UPLC-MS/MS for Determination of 36 Monomers of Alkylphenol Ethoxylates in Tea

**Lin Qin [1], Qin Yujie, Sun Hezhi, Wang Xinru, Yang Mei, Zhang Xinzhong, Zhou Li [1]*, Luo Fengjian[1]***

[1] Tea Research Institute, Chinese Academy of Agricultural Sciences, Hangzhou 310008, China; linqin202302@163.com

* Correspondence: lizhou@tricaas.com, +86-571-86653171; lfj@tricaas.com, +86-571-86650624

## Contents

**Table S1.** Chemical structures NPEOs and OPEOs.

**Table S2.** Validated parameters of $APEO_{3\text{-}20}$ in spiked tea samples.

**Table S3.** Mass spectrometric parameters for monitoring $OPEO_{3\text{-}20}$ and $NPEO_{3\text{-}20}$.

**Figure S1.** Full-scan mass spectra of (A) OPEO (9-10) ($n_{EO}$ = 3-15), (B) OPEO (16) ($n_{EO}$ = 9-20), (C) NPEO (4) ($n_{EO}$ = 9-11), (D) NPEO (9-10) ($n_{EO}$ = 3-15), and (E) NPEO (14) ($n_{EO}$ = 6-20) in 5 mg/kg standards.

**Figure S2.** Chromatograms of (A) $NPEO_{17}$ and $NPEO_{19}$ combined two ammonium ions, and (B) $NPEO_{17}$ and $NPEO_{19}$ combined one ammonium ion.

**Figure S3.** Multiple reaction-monitoring chromatograms of HPLC-MS/MS for blank tea samples spiked at the middle concentration of OPEOs and NPEOs.

**Table S1.** Chemical structures NPEOs and OPEOs.

| Analyte | Chemical structures |
| --- | --- |
| NPEOs | 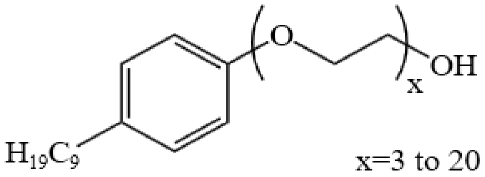  |
| OPEOs | 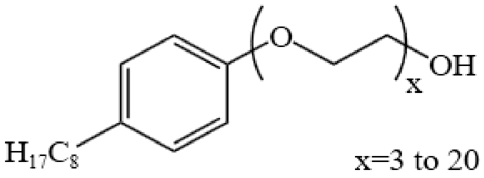  |

**Table S2.** Validated parameters of $APEO_{3\text{-}20}$ in spiked tea samples.

| Analyte | Spiked level (μg/kg) | Matrix standard curve | $R^2$ | Recovery % | RSD% (*n*=5) | LOD (μg/kg) | LOQ (μg/kg) | Analyte | Spiked level (μg/kg) | Matrix standard curve | $R^2$ | Recovery % | RSD% (*n*=5) | LOD (μg/kg) | LOQ (μg/kg) |
|---|---|---|---|---|---|---|---|---|---|---|---|---|---|---|---|
| $OPEO_3$ | 0.024 | y = 263254 x + 2715 | 0.9989 | 98.6 | 3.4 | 0.008 | 0.024 | $NPEO_3$ | 2.79 | y = 49852 x + 30818 | 0.9993 | 110.7 | 6.3 | 0.93 | 2.79 |
| | 0.24 | | | 83.0 | 2.8 | | | | 27.85 | | | 79.8 | 5.1 | | |
| | 0.47 | | | 88.4 | 6.9 | | | | 55.70 | | | 83.0 | 3.5 | | |
| $OPEO_4$ | 0.092 | y = 109203 x + 2934 | 0.9980 | 101.4 | 8.1 | 0.031 | 0.092 | $NPEO_4$ | 5.01 | y = 20380 x + 19496 | 0.9993 | 107.1 | 6.7 | 1.67 | 5.01 |
| | 0.92 | | | 87.0 | 3.1 | | | | 50.10 | | | 85.2 | 4.6 | | |
| | 1.84 | | | 92.8 | 6.0 | | | | 100.20 | | | 90.5 | 4.4 | | |
| $OPEO_5$ | 0.54 | y = 23913 x + 2570 | 0.9990 | 103.3 | 10.8 | 0.18 | 0.54 | $NPEO_5$ | 4.99 | y = 12188 x + 15110 | 0.9993 | 101.3 | 2.9 | 1.66 | 4.99 |
| | 5.37 | | | 88.7 | 4.3 | | | | 49.90 | | | 87.2 | 4.4 | | |
| | 10.73 | | | 93.7 | 5.8 | | | | 99.80 | | | 93.7 | 2.0 | | |
| $OPEO_6$ | 1.31 | y = 12685 x + 8309 | 0.9949 | 104.1 | 11.9 | 0.44 | 1.31 | $NPEO_6$ | 3.75 | y = 12123 x + 1432 | 0.9999 | 98.4 | 7.5 | 1.25 | 3.75 |
| | 13.13 | | | 87.8 | 4.8 | | | | 37.50 | | | 91.9 | 5.0 | | |
| | 26.26 | | | 89.2 | 5.0 | | | | 75.00 | | | 86.3 | 3.2 | | |
| $OPEO_7$ | 2.39 | y = 11890 x + 6313 | 0.9988 | 92.2 | 5.9 | 0.80 | 2.39 | $NPEO_7$ | 3.86 | y = 8074 x + 10458 | 0.9982 | 99.5 | 2.4 | 1.29 | 3.86 |
| | 23.88 | | | 86.6 | 2.9 | | | | 38.60 | | | 92.3 | 4.6 | | |
| | 47.76 | | | 91.9 | 4.9 | | | | 77.20 | | | 84.9 | 4.3 | | |
| $OPEO_8$ | 3.29 | y = 263254 x + 2715 | 0.9989 | 95.0 | 4.6 | 1.10 | 3.29 | $NPEO_8$ | 3.54 | y = 10035 x + 4451 | 0.9997 | 104.5 | 4.3 | 1.18 | 3.54 |
| | 32.90 | | | 91.2 | 4.9 | | | | 35.35 | | | 83.0 | 6.2 | | |
| | 65.80 | | | 89.0 | 7.6 | | | | 70.70 | | | 87.9 | 6.6 | | |
| $OPEO_9$ | 5.16 | y = 8207 x + 10957 | 0.9987 | 97.5 | 4.2 | 1.72 | 5.16 | $NPEO_9$ | 4.29 | y = 7291 x + 6178 | 0.9996 | 100.4 | 4.1 | 1.43 | 4.29 |
| | 51.61 | | | 88.7 | 4.2 | | | | 42.85 | | | 89.6 | 6.4 | | |
| | 103.22 | | | 90.9 | 5.7 | | | | 85.70 | | | 88.3 | 4.6 | | |
| $OPEO_{10}$ | 5.83 | y = 8009 x + 11700 | 0.9987 | 93.5 | 5.5 | 1.94 | 5.83 | $NPEO_{10}$ | 4.64 | y = 7108 x + 3919 | 0.9997 | 100.1 | 6.8 | 1.55 | 4.64 |
| | 58.26 | | | 90.7 | 5.7 | | | | 46.35 | | | 86.7 | 5.3 | | |
| | 116.52 | | | 93.3 | 3.9 | | | | 92.70 | | | 92.2 | 5.8 | | |
| $OPEO_{11}$ | 6.27 | y = 7754 x + 15942 | 0.9973 | 98.5 | 7.7 | 2.09 | 6.27 | $NPEO_{11}$ | 4.13 | y = 6815 x + 5285 | 0.9992 | 98.6 | 7.8 | 1.38 | 4.13 |
| | 62.69 | | | 87.4 | 5.3 | | | | 41.25 | | | 89.4 | 6.6 | | |
| | 125.38 | | | 90.8 | 6.5 | | | | 82.50 | | | 89.2 | 4.1 | | |
| $OPEO_{12}$ | 5.86 | y = 8177 x + 15620 | 0.9974 | 93.3 | 5.6 | 1.95 | 5.86 | $NPEO_{12}$ | 3.74 | y = 6052 x + 3328 | 0.9991 | 96.9 | 3.9 | 1.25 | 3.74 |
| | 58.57 | | | 88.0 | 7.4 | | | | 37.35 | | | 82.0 | 5.6 | | |
| | 117.13 | | | 91.9 | 6.5 | | | | 74.70 | | | 92.9 | 3.6 | | |
| $OPEO_{13}$ | 5.36 | y = 9044 x + 10924 | 0.9986 | 95.3 | 5.4 | 1.79 | 5.36 | $NPEO_{13}$ | 3.06 | y = 3212 x + 1279 | 0.9999 | 99.6 | 13.0 | 1.02 | 3.06 |
| | 53.62 | | | 85.6 | 5.3 | | | | 30.55 | | | 88.1 | 6.4 | | |
| | 107.24 | | | 93.2 | 8.9 | | | | 61.10 | | | 91.2 | 5.4 | | |
| $OPEO_{14}$ | 5.20 | y = 263254 x + 2715 | 0.9989 | 93.6 | 5.5 | 1.73 | 5.20 | $NPEO_{14}$ | 2.46 | y = 1878 x + 660 | 0.9998 | 98.3 | 4.2 | 0.82 | 2.46 |
| | 52.00 | | | 84.3 | 4.3 | | | | 24.60 | | | 79.1 | 3.9 | | |
| | 104.00 | | | 87.8 | 7.4 | | | | 49.20 | | | 89.4 | 6.2 | | |

| | | | | | | | | | | | | | | | |
|---|---|---|---|---|---|---|---|---|---|---|---|---|---|---|---|
| OPEO$_{15}$ | 3.81 | y = 9334 x + 6486 | 0.9992 | 89.2 | 6.9 | 1.27 | 3.81 | NPEO$_{15}$ | 1.48 | y = 5867 x + 233 | 0.9999 | 107.2 | 9.8 | 0.49 | 1.48 |
| | 38.07 | | | 83.8 | 7.0 | | | | 14.80 | | | 85.5 | 4.0 | | |
| | 76.13 | | | 88.8 | 6.6 | | | | 29.60 | | | 91.3 | 5.2 | | |
| OPEO$_{16}$ | 2.24 | y = 5616 x + 163 | 0.9999 | 89.0 | 8.1 | 0.75 | 2.24 | NPEO$_{16}$ | 0.86 | y = 8816 x + 1169 | 0.9993 | 100.1 | 8.0 | 0.29 | 0.86 |
| | 22.43 | | | 72.6 | 3.8 | | | | 8.55 | | | 78.4 | 12.8 | | |
| | 44.85 | | | 82.9 | 14.5 | | | | 17.10 | | | 90.4 | 11.9 | | |
| OPEO$_{17}$ | 1.27 | y = 12949 x + 552 | 0.9999 | 83.2 | 9.3 | 0.42 | 1.27 | NPEO$_{17}$ | 0.61 | y = 8572 x + 1370 | 0.9978 | 90.9 | 16.9 | 0.20 | 0.61 |
| | 12.75 | | | 78.5 | 7.1 | | | | 6.05 | | | 84.1 | 11.9 | | |
| | 25.49 | | | 88.8 | 2.1 | | | | 12.10 | | | 83.3 | 11.6 | | |
| OPEO$_{18}$ | 0.56 | y = 32169 x - 1062 | 0.9999 | 77.8 | 6.7 | 0.17 | 0.56 | NPEO$_{18}$ | 0.43 | y = 5821 x + 293 | 0.9997 | 105.2 | 15.4 | 0.14 | 0.43 |
| | 5.56 | | | 78.9 | 2.4 | | | | 4.25 | | | 87.5 | 3.7 | | |
| | 11.12 | | | 81.6 | 7.2 | | | | 8.50 | | | 91.0 | 7.3 | | |
| OPEO$_{19}$ | 0.63 | y = 23701 x + 1129 | 0.9995 | 76.6 | 12.5 | 0.21 | 0.63 | NPEO$_{19}$ | 0.27 | y = 9757 x + 228 | 0.9998 | 75.6 | 7.9 | 0.090 | 0.27 |
| | 6.33 | | | 70.3 | 4.8 | | | | 2.65 | | | 81.5 | 2.4 | | |
| | 12.66 | | | 77.9 | 3.6 | | | | 5.30 | | | 87.2 | 2.0 | | |
| OPEO$_{20}$ | 0.17 | y = 23593 x + 611 | 0.9988 | 66.9 | 1.5 | 0.057 | 0.17 | NPEO$_{20}$ | 0.16 | y=23166 x + 375 | 0.9999 | 74.2 | 13.3 | 0.053 | 0.16 |
| | 1.70 | | | 61.8 | 7.5 | | | | 1.62 | | | 66.9 | 13.6 | | |
| | 3.40 | | | 72.1 | 3.1 | | | | 3.24 | | | 62.9 | 16.9 | | |

**Table S3.** Mass spectrometric parameters for monitoring $OPEO_{3-20}$ and $NPEO_{3-20}$.

| Analyte | Retention time | Precursor ion (m/z) | Product Ion (m/z) | Cone Voltage (eV) | Collision voltage ( V) | Analyte | Retention time | Precursor ion (m/z) | Product Ion (m/z) | Cone Voltage (eV) | Collision voltage ( V) |
|---|---|---|---|---|---|---|---|---|---|---|---|
| $OPEO_3$ | 6.11 | 356.3 | 227.2* | 15 | 10 | $NPEO_3$ | 6.77 | 370.1 | 121.3* | 30 | 30 |
| | | | 121.2 | | 15 | | | | 227.1 | | 10 |
| $OPEO_4$ | 6.11 | 400.3 | 271.2 | 15 | 25 | $NPEO_4$ | 6.77 | 414.1 | 397.2* | 30 | 10 |
| | | | 383.2* | | 10 | | | | 270.9 | | 15 |
| $OPEO_5$ | 6.11 | 444.3 | 427.3* | 15 | 14 | $NPEO_5$ | 6.77 | 458.1 | 315.0* | 30 | 15 |
| | | | 315.3 | | 16 | | | | 441.6 | | 10 |
| $OPEO_6$ | 6.10 | 488.3 | 89.1* | 22 | 29 | $NPEO_6$ | 6.76 | 502.2 | 133.1* | 30 | 18 |
| | | | 471.4 | | 15 | | | | 485.4 | | 15 |
| $OPEO_7$ | 6.08 | 532.4 | 89.1* | 25 | 30 | $NPEO_7$ | 6.74 | 546.3 | 529.1 | 30 | 15 |
| | | | 515.3 | | 15 | | | | 132.9* | | 28 |
| $OPEO_8$ | 6.06 | 576.4 | 89.1* | 25 | 30 | $NPEO_8$ | 6.71 | 590.3 | 291.1 | 30 | 26 |
| | | | 133.2 | | 25 | | | | 133.3* | | 30 |
| $OPEO_9$ | 6.04 | 620.4 | 89.1* | 23 | 40 | $NPEO_9$ | 6.68 | 634.4 | 291.1 | 30 | 30 |
| | | | 133.2 | | 28 | | | | 133.3* | | 30 |
| $OPEO_{10}$ | 6.01 | 664.4 | 89.1 | 25 | 40 | $NPEO_{10}$ | 6.65 | 678.5 | 291.1* | 30 | 30 |
| | | | 133.2* | | 28 | | | | 133.3 | | 30 |
| $OPEO_{11}$ | 5.99 | 708.5 | 89.1* | 25 | 40 | $NPEO_{11}$ | 6.62 | 722.4 | 177.1 | 50 | 30 |
| | | | 133.2 | | 30 | | | | 133.2* | | 30 |
| $OPEO_{12}$ | 5.97 | 752.5 | 89.1* | 25 | 40 | $NPEO_{12}$ | 6.60 | 766.6 | 290.6 | 30 | 30 |
| | | | 133.2 | | 30 | | | | 132.7* | | 30 |
| $OPEO_{13}$ | 5.94 | 796.6 | 89.1* | 25 | 40 | $NPEO_{13}$ | 6.56 | 810.6 | 793.2 | 30 | 20 |
| | | | 277.2 | | 30 | | | | 133.4* | | 38 |
| $OPEO_{14}$ | 5.92 | 840.6 | 89.1* | 25 | 40 | $NPEO_{14}$ | 6.54 | 854.6 | 177 | 30 | 30 |
| | | | 133.2 | | 35 | | | | 133.0* | | 40 |
| $OPEO_{15}$ | 5.90 | 884.7 | 89.1* | 25 | 40 | $NPEO_{15}$ | 6.51 | 898.7 | 291.1 | 30 | 30 |
| | | | 133.2 | | 40 | | | | 133.2* | | 40 |
| $OPEO_{16}$ | 5.88 | 473.2 | 89.0* | 30 | 30 | $NPEO_{16}$ | 6.48 | 479.8 | 89.3* | 30 | 24 |
| | | | 408.8 | | 10 | | | | 133.3 | | 20 |
| $OPEO_{17}$ | 5.85 | 495.3 | 89.1* | 10 | 30 | $NPEO_{17}$ | 6.46 | 986.6 | 88.9* | 28 | 60 |
| | | | 133 | | 30 | | | | 133.0 | | 20 |
| $OPEO_{18}$ | 5.83 | 517.3 | 89.1* | 10 | 30 | $NPEO_{18}$ | 6.43 | 523.8 | 133.0 | 10 | 22 |
| | | | 133.2 | | 30 | | | | 89.1* | | 30 |
| $OPEO_{19}$ | 5.81 | 539.4 | 89.3* | 10 | 30 | $NPEO_{19}$ | 6.40 | 1074.7 | 89.1* | 20 | 50 |
| | | | 133.2 | | 30 | | | | 133.2 | | 50 |
| $OPEO_{20}$ | 5.79 | 561.4 | 89.5* | 10 | 35 | $NPEO_{20}$ | 6.34 | 568.0 | 132.9 | 20 | 27 |
| | | | 132.6 | | 20 | | | | 89.0* | | 30 |

* indicates quantitative ion.

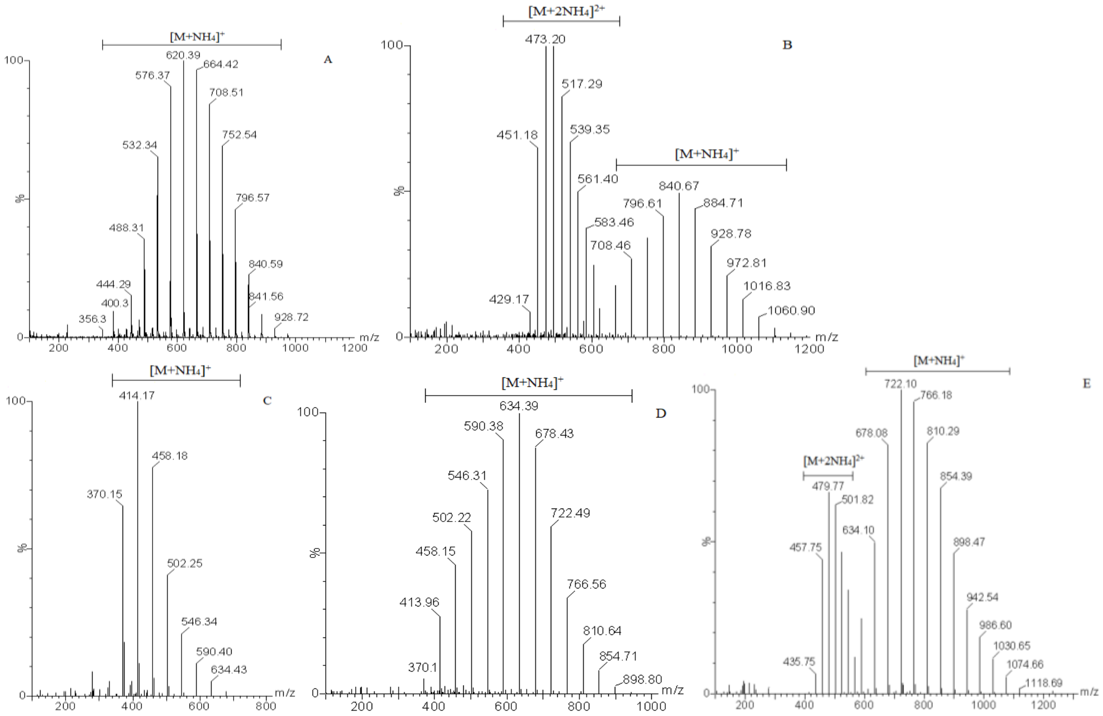


**Figure S1.** Full-scan mass spectra of (A) OPEO (9-10) ($n_{EO}$ = 3-15), (B) OPEO (16) ($n_{EO}$ = 9-20), (C) NPEO (4) ($n_{EO}$ = 9-11), (D) NPEO (9-10) ($n_{EO}$ = 3-15), and (E) NPEO (14) ($n_{EO}$ = 6-20) in 5 mg/kg standards.

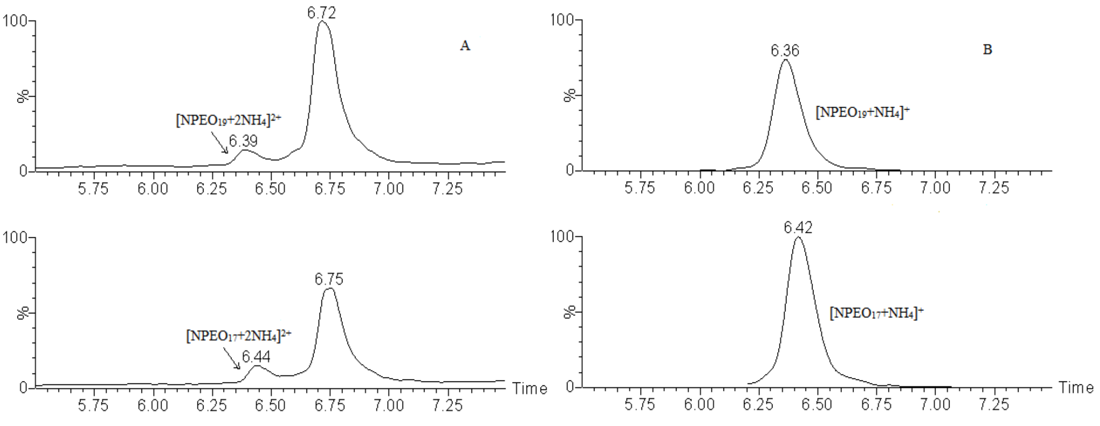


**Figure S2.** Chromatograms of (A) $NPEO_{17}$ and $NPEO_{19}$ combined two ammonium ions, and (B) $NPEO_{17}$ and $NPEO_{19}$ combined one ammonium ion.

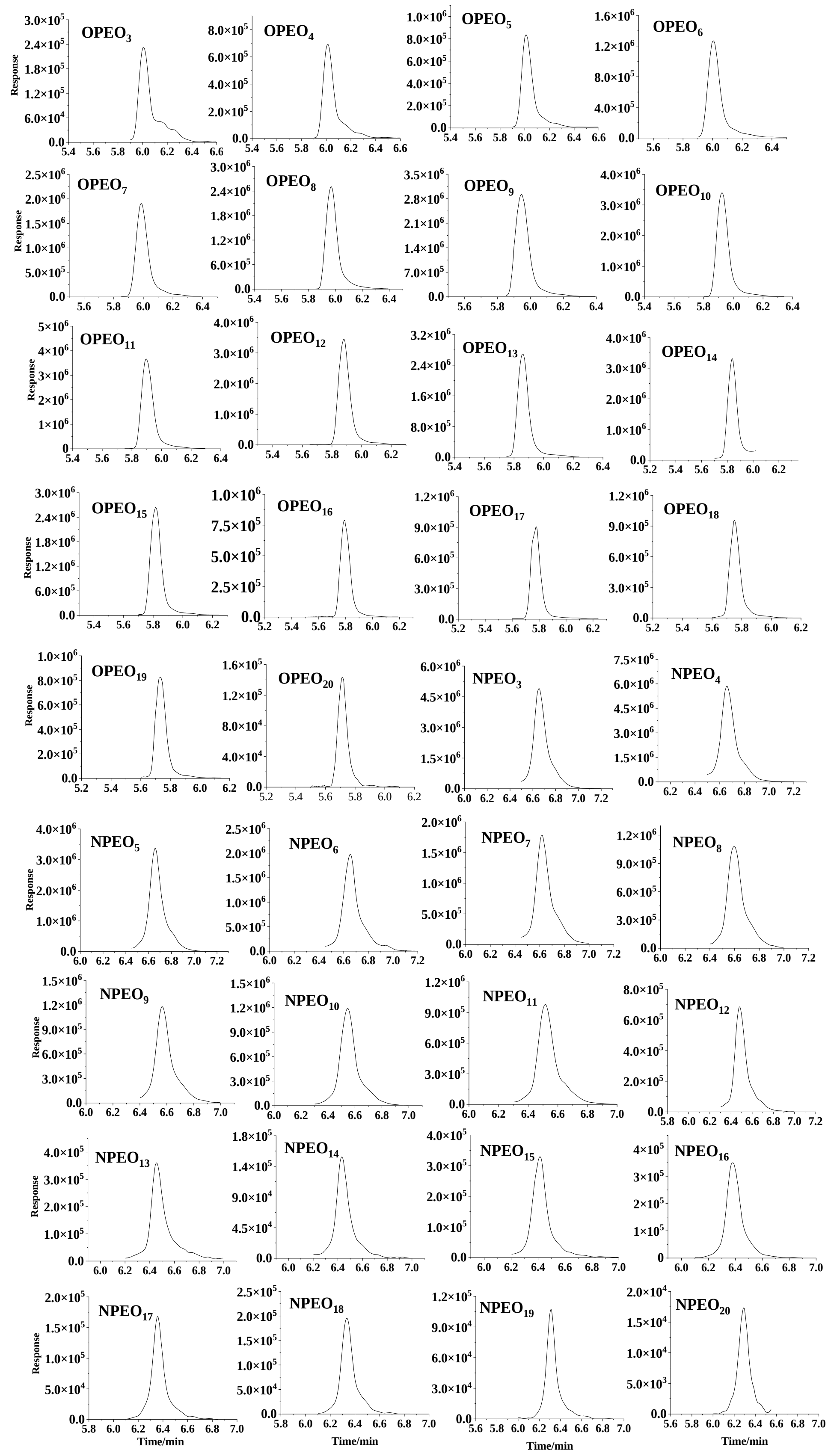


**Figure S3.** Multiple reaction-monitoring chromatograms of HPLC-MS/MS for blank tea samples spiked at the middle concentration of OPEOs and NPEOs.
